# A Comprehensive Review of the Fabella Bone

**DOI:** 10.7759/cureus.2736

**Published:** 2018-06-05

**Authors:** Dominic Dalip, Joe Iwanaga, Rod J Oskouian, R. Shane Tubbs

**Affiliations:** 1 Seattle Science Foundation, Seattle, USA; 2 Swedish Neuroscience Institute, Seattle, USA; 3 Neurosurgery, Seattle Science Foundation, Seattle, USA

**Keywords:** sesamoid, knee pain, gastrocnemius, femoral condyle, fabellectomy, shock wave therapy, anatomy, variations

## Abstract

The fabella is a sesamoid bone that is embedded in the lateral head of the gastrocnemius muscle and often articulates directly with the lateral femoral condyle. It is present in 10-30% of the general population with a higher incidence in Asians. The fabella can lead to various pathologies such as fabella pain syndrome and common fibular nerve palsy. Conservative treatment involves physical therapy or injecting local anesthetics or steroids around this bone. However, if symptoms persist, then a fabellectomy can be performed. Physicians should be aware of the fabella bone and the multiple pathologies associated with it in order to provide the best treatment and management for patients.

## Introduction and background

**Figure 1:**
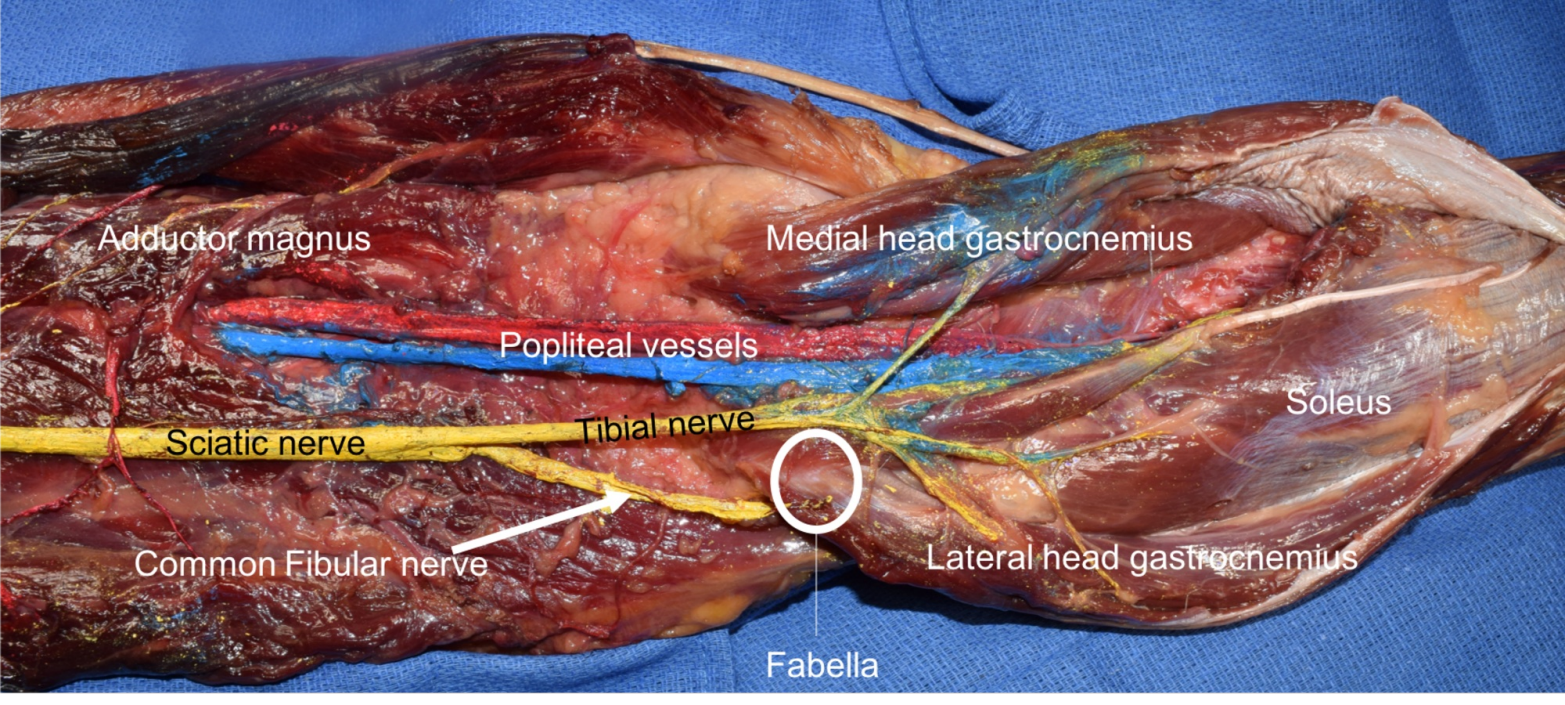
Posterior view of the distal left thigh and proximal leg. Note the fabella as seen within the proximal tendon of the lateral head of the gastrocnemius.

**Figure 2:**
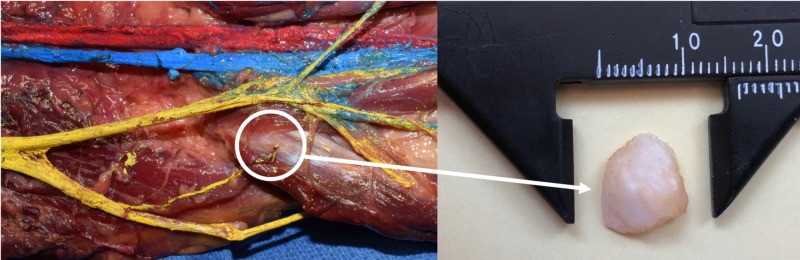
Zoomed in view of Figure 1 noting the fabella (circle). After removal (right image), the fabella was found to be bony in nature and approximately 1 cm in diameter.

The patella is the largest and most well-known sesamoid bone. Other normally found sesamoid bones are seen in the hands and feet. A relatively unknown sesamoid bone of the leg is the fabella (Figures 1, 2). This sesamoid bone is embedded in the lateral head of the gastrocnemius muscle and often articulates directly with the lateral femoral condyle. The fabella’s main function is thought to be stabilization of the medial femoral condyle and the fabella complex, which is made up of the plantaris and gastrocnemius muscles and the arcuate, fabellofibular, fabellopopliteal, and oblique popliteal ligaments [1-3].

Various pathologies have been attributed to the presence of a fabella such as fabella pain syndrome, common fibular (CF) nerve palsy, fabella fracture, and Popliteal Artery Entrapment Syndrome (PAES) [4,5]. Therefore, the aim of this paper is to review the anatomy of the fabella and its related pathologies and treatment.

## Review

Anatomy

The fabella usually ranges from 5 mm to 20 mm in diameter (Figure 2) and occupies about 26% of the length of the CF nerve across the length of the lateral gastrocnemius. It is found in approximately 10% to 30% of the population and it occurs bilaterally in approximately 80% of cases [1,6,7]. In the Asian population, the fabella has a reported prevalence of 25% to 87% [2, 3, 7, 8]. The rates of a bilateral fabella were almost identical in Asian and non-Asian populations due to the differences in radiological and anatomical studies [3]. In radiological studies, the prevalence of the fabella was similar for both age and sex in the general population [9]. Males had a fabella frequency of 21.2 % whereas females had a frequency of 27.2% and that there were no significant sex-based differences [1]. A gross anatomical study found that the occurrence of the fabella was 68.6% [3,7]. Physicians can mistake the fabella for loose bodies or osteophytes which are usually asymptomatic in patients.

The fabella can be bony or cartilaginous. From 150 fabellae that were studied, 72 were cartilaginous and 27 were bony [7]. These results suggest that the fabella is formed by endochondral ossification. The consensus is that a sesamoid bone is formed from mechanical stress on a tendon [10-12]. The fabellofibular ligament and the fabella are formed from an evolutionary standpoint where humans moved from a quadrupedal to a bipedal posture [13].

A total of 102 knees of 51 cadavers were examined to determine the morphology of the fabella and the CF nerve in the popliteal region [3]. The fabella occupies about 26% of the length of the CF nerve across the length of the lateral head of the gastrocnemius [2, 3]. This study found that the CF nerve adjacent to the fabella was wider and thinner compared to the proximal fabella in cases where the nerve was passing posterior and lateral to the fabella. In other cases where the CF nerve passed medial to the fabella, or when the fabella was absent, there were no differences in the size of the nerve as it passed adjacent to the fabella.

In cases of CF palsy, 20.8% of patients had the nerve located posterior to the fabella [8]. There were only a few cases of CF palsy in obese populations [14]. Therefore, some have posited that the less subcutaneous fat there is, the more prone the CF nerve is to compression by the fabella.

Pathology and diagnosis

Fabella pain syndrome should be considered as a differential diagnosis when a patient presents with persistent posterolateral knee pain, which could also be due to meniscal tears, lateral ligament instability, Baker’s cyst, and proximal tibiofibular joint hypomobility [3, 15-20]. Patients with Fabella pain syndrome usually complain that the posterolateral knee pain is worse on fully extending the legs at the knee joint [21].

Fracture of the fabella is a rare entity but can happen due to direct trauma or chronic stress forces [5]. Three cases of stress fracture of the fabella were reported following total knee arthroplasty [18, 22]. These fractures varied from four months to nine years after surgery. Patients presented with swelling and pain of the posterolateral aspect of the knee. CT or MRI confirms the diagnosis and guides therapy.

The fabella is a cause of CF nerve palsy [14]. Seven cases were reviewed where the fabella compressed the CF nerve [23]. Three of the cases were treated surgically with fabellectomy and showed dramatic improvement in symptoms as soon as the first postoperative day. The other four cases were managed conservatively and showed improvement three to four days after treatment. Overall, improvement was seen between two weeks to two months after treatment.

PAES is a term that was first introduced by Love and Whelan in 1965 [24]. This syndrome occurs when the popliteal artery is compressed by musculotendinous structures in the popliteal fossa. Recurrent compression of the popliteal artery can lead to intimal damage, distal embolization, thrombosis, post-stenotic dilation and true aneurysms. The first known case of fabella pain syndrome with PAES was in a patient presented with intermittent claudication and severe knee osteoarthritis due to the fabella compressing the popliteal artery [24]. This was diagnosed using CT angiography which showed left popliteal artery occlusion without development of a collateral circulation. In this case, the treatment was fabella resection with revascularization of the popliteal artery. A better understanding of the anatomy of the knee and its variations is important in diagnosing and treating patients with pathology of this area [25-33].

Treatment

Fabella pain syndrome can be treated with physical therapy, injection of local anesthetics or steroids near the site, radial extracorporeal shock wave therapy (rESWT) or fabellectomy [6]. Physical therapy entails the patient be placed in a prone position with the legs supported at an angle of 30 degrees flexion [15]. Sustained pressure on the skin and deeper soft tissue is then applied along the directions of mobility restrictions incorporating the gastric-soleus complex and then the lateral head of the gastrocnemius is gently stretched. This technique is performed for approximately three minutes. The patient usually experiences immediate pain-free motions with flexion of the knee of up to 120 degrees.

rESWT entails three thousand shock waves being delivered at a frequency of 12 Hz. This procedure can be performed at two-week intervals for a total of one to four times. The mechanism of rESWT involves destruction of the unmyelinated sensory nerves, hyperstimulation analgesic effect, and neovascularization in degenerated tissues. In one series, post treatment, patients noticed a sudden decrease in pain intensity: in three cases, pain intensity ranged from an eight to a one; and in one case, pain intensity ranged from a four to a zero. These decreases in pain intensity scale remained at a two-month follow-up clinical visit.

## Conclusions

The fabella is a variant sesamoid bone that can lead to various pathologies such as fabella pain syndrome, CF nerve palsy, and popliteal entrapment syndrome. It is important for physicians to be aware of the fabella because it can be mistaken for osteophytes or loose structures that the surgeon may explore and that may put the patient at risk of neurovascular injuries. Also, the fabella is a rare cause of persistent posterolateral knee pain that physicians should be aware of as a differential diagnosis. A better understanding of the anatomy of the knee and its variations is important in diagnosing and treating patients with pathology of this area.
